# Editorial: Lung microbiome in health and disease

**DOI:** 10.3389/fphar.2025.1565849

**Published:** 2025-02-14

**Authors:** Kurtis Francis Budden, Luigina Romani

**Affiliations:** ^1^ Priority Research Centre for Healthy Lungs and Immune Health Research Program, School of Biomedical Sciences and Pharmacy, The University of Newcastle and Hunter Medical Research Institute, Newcastle, NSW, Australia; ^2^ Department of Medicine and Surgery, University of Perugia, Perugia, Italy; ^3^ Research Center San Raffaele, Sulmona, Italy

**Keywords:** microbiota, immunity, lung diseases, immunomodulation, metabolites

The discovery of a complex network of microbial populations within the lung has transformed it into a significant research focus. The lung microbiome ecology is much more dynamic and transient than those displayed by other sites such as the skin, intestine, or oral cavity (Budden et al., 2019). In acute and chronic respiratory diseases, there is a notable transition in the microbiome composition and distribution due to modifications in the rates of microbe immigration, elimination, and reproduction, which in turn impacts pathological developments (Budden et al., 2019). Thus, deciphering the relationships between host and resident or colonizing microbes is essential for proper diagnosis, prognosis, management, and treatment of respiratory conditions. This Research Topic discusses recent discoveries highlighting how deciphering host-microbe interactions may increase our understanding of the dynamics of pneumonia, lung cancer, and chronic obstructive pulmonary disease (COPD), as well as the effects of inhalation therapy on the local microbial community

Xi et al. characterized the lower respiratory tract (LTR) microbiota in children’s refractory *Mycoplasma pneumoniae* pneumonia before and after the COVID-19 pandemic. Despite no significant differences in the α- and β-diversity, some microbial species including *Trichoderma_citrinoviride, Canine_mastadenovirus_A, Ralstonia_pickettii, Lactococcus_lactis, Pseudomonas_aeruginosa* and *Mycoplasmoides pneumonia* were more abundant in the LRT microbiota in the post-COVID groups and positively correlated with infection severity and impaired immunity.

In lung carcinogenesis, Zhai et al. describes the interplay between bacteria and their metabolites in different early-stage lung cancer specimens that could be used as potential biomarkers to guide future therapeutic strategies. Different microbial richness was found in the different specimens, with a lower richness characterizing the early-stage adenocarcinoma. Bacterial species were also different in the different specimens, with *Ralstonia* associated with early lung adenocarcinoma, and *Feacalibacterium* and *Blautia* associated with ground glass nodules that had not progressed to solid nodules. *Akkermansia, Escherichia-shigella,* and *Klebsiella* were instead associated with lung squamous carcinoma. Metabolites differed in the adenocarcinomas *versus* the squamous carcinoma, likely reflecting differing metabolic activities in energy and glutathione metabolism, respectively.

Two articles focus on the influence of long-term inhaled corticosteroid and modulation of the respiratory microbiota in COPD. Yue et al. employed a single-center retrospective cohort study to compare alterations in airway function and the sputum microbial community structure between COPD patients who had undergone either long-term or short-term treatment with inhaled corticosteroid. The study found a significantly altered β-diversity of the microbial community structure in the sputum of patients on long-termcorticosteroids therapy, with an increased abundance of *Abiotrophia, Schaalia, Granulicatella, Mogibacterium, Sphingobium*, and *Paraeggerthella* bacterial genera compared to short-term corticosteroids. Some of these were positively correlated with the eosinophil %. Confirming the role of the oral microbiota in COPD clinical dynamics, Hua et al. found that oral probiotic *Lactobacillus rhamnosus* GG administration significantly delayed exacerbation in patients with moderate-to-very severe COPD, an effect similarly obtained upon influenza*-S. pneumoniae* vaccination. Finally, Garaci et al. discuss the intricate inter-microbe association networks that comprise true mutualistic or antagonistic direct or indirect relationships in the respiratory tract. In particular, the analysis of the tripartite interaction of bacteria, fungi and the mammalian host has highlighted how the understanding of the metabolic and immune significance of their interaction could be valuable in development of novel druggable targets in disease treatments.

Overall, the articles included in this Research Topic highlight important aspects associated with the microbiota dynamics in the respiratory tract that help paving the way for the development of microbial based therapeutics approaches.
